# Factors affecting ENSO predictability in a linear empirical model of tropical air-sea interactions

**DOI:** 10.1038/s41598-020-60371-1

**Published:** 2020-03-03

**Authors:** Harun A. Rashid

**Affiliations:** Climate Science Centre, CSIRO Oceans and Atmosphere, Aspendale, VIC 3195 Australia

**Keywords:** Climate sciences, Ocean sciences

## Abstract

Understanding and extending the predictability of El Niño‒Southern Oscillation (ENSO) has been an important research topic because of ENSO’s large influence on global weather and climate. Here, we develop an empirical model of tropical atmosphere-ocean interactions that has high ENSO prediction skill, comparable to the skills of well performing dynamical models. The model is used to investigate the effects of the main atmosphere-ocean interaction processes—thermocline and zonal wind feedbacks and zonal wind forcing—on its ENSO predictability. We find that all these processes significantly affect ENSO predictability and extend the predictability limit by up to four months, with the largest effect coming from the thermocline feedback followed by the total zonal wind forcing. The other processes with progressively smaller effects are the external zonal wind forcing and zonal wind feedback. The two most influential processes, however, affect ENSO predictability in the VAR model differently. The thermocline feedback improves the forecast skill by predominantly maintaining the correct phase, whereas the total zonal wind forcing improves the skill by maintaining the correct amplitude of the forecast ENSO events. This result suggests that the dynamical seasonal prediction models must have good representations of the major ENSO processes to make skilful ENSO predictions.

## Introduction

El Niño‒Southern Oscillation (ENSO) is the dominant mode of tropical interannual climate variability. It arises from atmosphere-ocean interactions in the tropical Pacific and is associated with large anomalies of sea-surface temperatures, zonal winds and rainfall. ENSO also influences seasonal-to-interannual climate variations in many remote regions through atmospheric teleconnections^1^. Predicting ENSO is therefore an important part of routine seasonal predictions prepared by climate prediction centres around the world. In recent decades, dynamical seasonal prediction models have shown a great promise for providing skilful ENSO forecasts at interannual time scales^2^; however, progress towards this goal, while significant, is still limited, partly because of the presence of systematic biases in the model simulated climate. Statistical prediction models, derived from observational data, also have long history of ENSO prediction; these models are also limited because of shortness of observational records, as well as their inability to capture the full complexity of a coupled atmosphere-ocean system^3^.

Despite their limitations, statistical or empirical models are still able to represent (albeit in a simplified way) many of the major atmosphere-ocean interaction processes characterizing ENSO. A carefully constructed empirical model can estimate the main ENSO processes directly from observational data, thus avoiding the problem arising from dynamical models’ systematic biases. Many authors have recently used empirical models of various forms for ENSO forecasting and diagnostic studies^3–8^. The relative simplicity of these models, compared to the general circulation models (GCMs) used for dynamical predictions, enables their use in a variety of process-based investigations of ENSO. Such a model, ideally with good ENSO prediction skills, can similarly be used to investigate the processes that may significantly affect ENSO predictability.

Many studies have elucidated the major ENSO processes using observations^9,10^ and a hierarchy of dynamical models, ranging from simple conceptual models to sophisticated GCMs^11–13^. The main conceptual picture that has emerged over the years may be described as follows^14,15^: Westerly wind anomalies, associated with anomalous deep convections in the central equatorial Pacific, force zonal current anomalies and thermocline depth anomalies. These then affect the SST anomalies in the central-eastern and eastern Pacific, respectively, through zonal advections and subsurface wave dynamics. These time-lagged changes in eastern Pacific SSTs forced by the central equatorial Pacific zonal wind anomalies may be referred to as the zonal wind forcing^15^. The eastern Pacific SST anomalies in turn feedback on the central Pacific westerly anomalies, thus completing the so-called Bjerknes feedback loop. The zonal wind forcing contributed by the SST-driven anomalies (i.e., the component due to zonal wind feedback) combines with that from atmospheric process-driven zonal wind anomalies (i.e., external zonal wind forcing) to form the total zonal wind forcing. The local Ekman feedback also contributes positively to the ENSO-related SST anomaly growth^16^. In addition to these tropical Pacific processes, processes arising from the extratropics and other ocean basins can also influence ENSO dynamics, but the main aspects of the latter can be modelled incorporating only the tropical Pacific processes.

In this work, we develop an empirical model of observed tropical Pacific atmosphere-ocean interactions, guided by the above conceptual picture of ENSO processes, to investigate the factors affecting ENSO predictability. Many previous studies have used empirical models for understanding ENSO properties^4–8,17–21^. However, most of these models are either first-order (i.e., single time-level) multi-variable models^4,7,8^ or higher-order (i.e., multiple time levels) single variable models^17,20^. Only one of these studies used a higher-order, multi-variable model^21^ and a couple of them included quadratic nonlinearity in their models^20,21^. These latter studies showed that while including nonlinearity helps capture the nonlinear aspects of ENSO (e.g., amplitude and duration asymmetries), it doesn’t necessarily improve the models’ ENSO prediction skill. Our aim here is to develop a higher-order multi-variable model, using the vector autoregressive (VAR) framework^22,23^, with high ENSO predictability and use it to investigate the effects of various air-sea interaction processes on ENSO predictability. Including multiple key variables as prognostics in the model and allowing their mutual interactions can increase the ENSO predictability in empirical models^7,8,21^. On the other hand, a higher-order model can also improve performance over its single time-level version (as will be demonstrated below; also, see Chen *et al*.^21^) by utilising the immediate past evolutions of the variables, in addition to their latest available state, in model estimation and predictions.

In this work, we use historical observations of tropical Pacific sea-surface temperature (SST), zonal wind stress (Taux) and the thermocline depth (approximated by the 20 °C isotherm depth, Z20) to build a number of VAR models. These are the key variables, also used in some other empirical models^4,7^, that participate in the Bjerknes feedback and determine bulk of the ENSO properties through their mutual interactions, as discussed above. After considering various candidate models, we use the one with the best ENSO prediction skill to (i) compare the model’s ENSO predictability limit with those from two representative dynamical seasonal prediction models from the North American multi-model ensemble (NMME): Canadian coupled model #2 (hereafter, CMC2-CanCM4) and the modified version of GFDL coupled model 2.1 (hereafter, GFDL-CM2p1) and (ii) estimate the effects of major atmosphere-ocean interaction processes, namely the thermocline and zonal wind feedbacks and the zonal wind forcing, on ENSO predictability.

## Results

### Model selection

**Figure 1:**
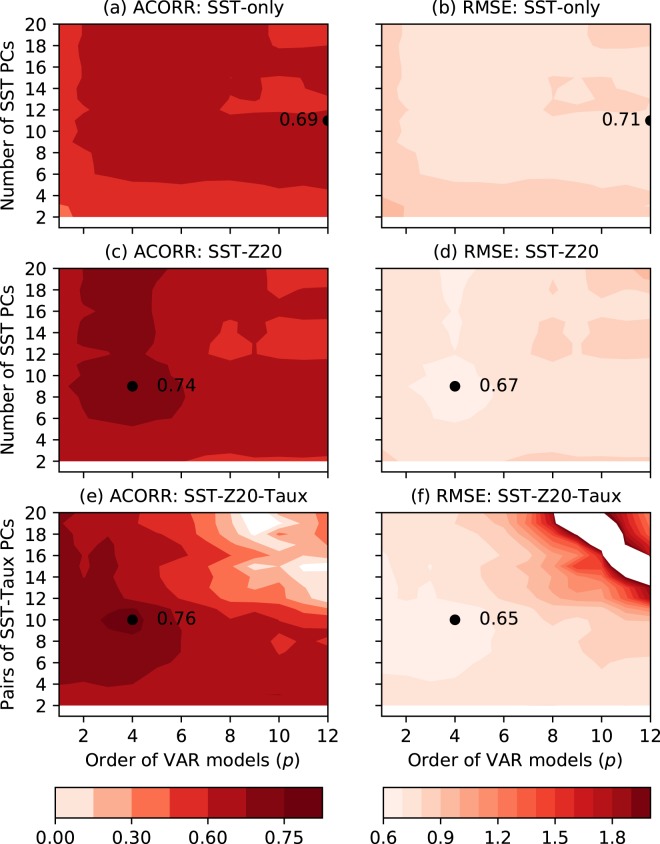
Anomaly correlation coefficients (ACORRs, left column) and root-mean squared errors (RMSEs, right column) of the six-month lead SST-PC1 forecasts as a function of model order (*p*) and the number of retained EOF modes (*m*). Results for three VAR models are shown: SST-only model (top row), SST-Z20 model (middle row) and SST-Z20-Taux model (bottom row). The black dots in the left and right columns indicate the maximum ACORR and minimum RMSE values, respectively, for different models. RMSE values are normalised by the observed SST-PC1 standard deviation and values larger than 2 are not plotted. The vertical axis shows the *number* of retained SST PCs in the top row, but the *pairs* of SST-Z20 PCs in the middle and bottom rows. Also, an additional Z20-PC2 is included in the SST-Z20-Taux model. Note that the colour shading for ACORR is not linear. The plot was created by python (version 2.7.6) and matplotlib (version 2.2.2) package^37^ (https://www.anaconda.com/distribution/).

The prediction skill of a VAR_*m*_(*p*) model varies depending on the number of EOF modes (i.e., principal component timeseries or PCs) retained (*m*) and the model order (*p*); see Methods for details of the estimation, prediction and verification of the VAR models used here. To select the best skilled model, we first built many VAR models for different values of *m* and *p* (using the training dataset for 1960–1990) and then evaluated their ENSO prediction skills on the verification dataset (1991–2017). Figure 1 shows the effects of variations of *p* and *m* on ENSO prediction skills at 6-month lead for three model configurations: SST-only, SST-Z20, and SST-Z20-Taux. For SST-only model (top row), the maximum anomaly correlation (ACORR) is 0.69 and the minimum root-mean-squared-error (RMSE) is 0.71, which occur at the same model order, *p* = 12, and number of retained modes, *m* = 11. Note that the maximum model order we examined is 12, so the highest prediction skill for this model could as well occur at an order higher than 12. Indeed, Chapman *et al*.^17^ found the maximum skill (ACORR ~0.71) at a model order of 15 using an SST-only model with 11 EOF modes, but this skill is very similar to what we find at order 12, so further increasing the order wouldn’t make the model more skilled. This consistency indicates the robustness of the SST-only model result, given that this other study used SST data for 1861–1980 for computing cross-validated skills.

For SST-Z20 model (middle row), the maximum ACORR is 0.74 and the minimum RMSE is 0.67 and these values occur at *p* = 4 and *m* = 10, comprising the 9 leading SST-PCs and the second leading mode of Z20 (hereafter, Z20-PC2). We included only Z20-PC2, because the other Z20 modes, including the leading one, do not increase the model’s ENSO prediction skill. Johnson *et al*.^8^ also noted a similar increase in their model’s ENSO prediction skill due to the inclusion of the second leading mode of tropical Pacific heat content anomalies. Z20-PC2 is closely related to the tropical Pacific warm water volume (WWV) or heat content anomalies; these are in turn highly correlated with the Niño-3 SST anomaly at a lead time of about 7 months^24^, thus making the SST-Z20 model more skilful than the SST-only model. This enhancement of ENSO prediction skill by including sub-surface information is consistent with the results of several previous studies, done using different types of empirical models^7,8,19,21^. For SST-Z20-Taux model (bottom row), the maximum ACORR (0.76) and the minimum RMSE (0.65) occur at *p* = 4 and *m* = 21 (with 10 leading pairs of SST and Taux PCs plus Z20-PC2); these are slightly better than the skill of the SST-Z20 model. However, the prediction skill gets substantially better with the addition of both Taux and Z20 to the SST-only model; these extra variables also help increase the model’s explained variance from 94% to 96%. This shows that the Z20 and Taux PCs included in the SST-Z20-Taux model Granger-cause the variability associated with SST-PC1^25,26^. All three higher-order VAR models also show increased skills compared to their first-order counterparts. For example, the ACORR value goes from 0.74 to 0.76 as the model order is increased from 1 to 4 for the SST-Z20-Taux model; for the other two models, these increases are even larger. The small improvement for the SST-Z20-Taux model over the SST-Z20 model is not found to be statistically significant, given the size of the verification dataset is not very large. The 95% confidence interval for the model’s ACORR skill (0.76) was estimated to be 0.71–0.81 using 100,000 bootstrap samples. However, as will be shown below, the slight superiority of the SST-Z20-Taux model holds also at other forecast leads (see Fig. 23 below), as well as when the prediction skill is estimated using the full cross-validation approach. Also, a main objective of this study is to understand the impacts of interactions between SST, Z20 and Taux on ENSO forecast skill. Therefore, we choose the 4-th order 21-component SST-Z20-Taux model [hereafter, VAR_21_(4)] as our overall best-skilled model.

**Figure 2:**
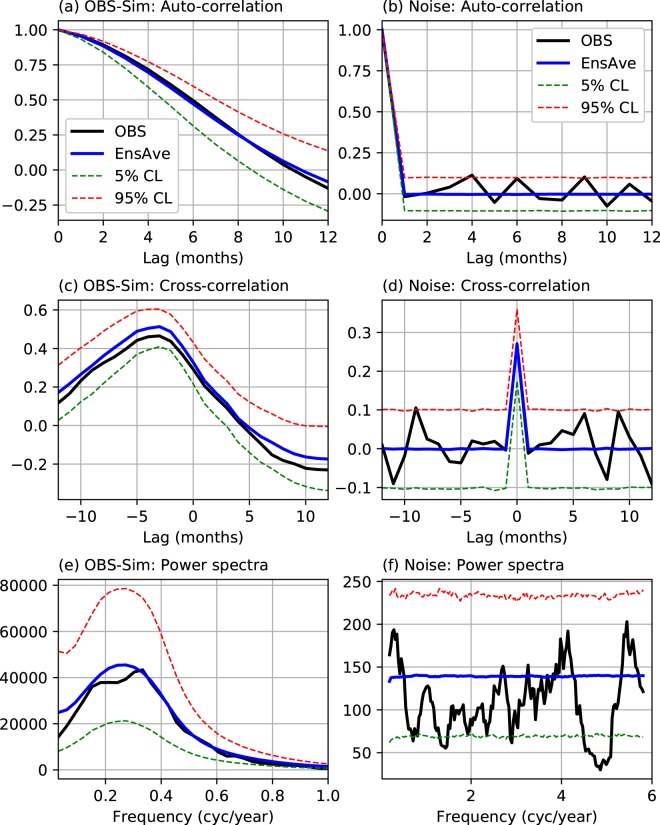
Auto-correlations of SST-PC1 (top row), cross-correlations between SST-PC1 and Taux-PC1 (middle row) and power spectra of SST-PC1 (bottom row) computed from observations (1960–1990) and a 100,000-year model simulation (left column) and from the model residuals and multivariate white noise with the same covariance matrix as for the residuals (right column). The black curves in the left and right columns show the statistics computed from observations and model residuals, respectively. The blue curves show the ensemble-averaged statistics and the dashed curves the 95% confidence interval. The entire simulation data from the SST-Z20-Taux model were divided into 3225 31-year long segments (equal to the length of the training data), and the statistics computed from individual segments were used to obtain the ensemble averages and their 95% confidence intervals. The plot was created by python (version 2.7.6) and matplotlib (version 2.2.2) package^37^ (https://www.anaconda.com/distribution/).

In addition to being the most skilful, the VAR_21_(4) model also explains the basic ENSO properties of the training dataset well, and the unexplained variations (i.e., residuals) are consistent with multivariate white noise, as required by this class of models. This is shown in Fig. 2 by comparing ENSO statistics (auto-correlation, cross-correlation and power spectrum) between observations and model simulations (left column) and between multivariate white noise and model residuals (right column). The simulated statistics were obtained from 1,200,000 months of simulation by the VAR_21_(4) model driven by white noise, the latter being drawn from a multi-variate normal distribution with the same covariance matrix as of the residuals (see the caption of Fig. 2 for more details). It is seen that the model simulated statistics match with the observed, and the residual statistics with the white noise statistics, at the 95% significance level, demonstrating the model’s goodness-of-fit.

**Figure 3:**
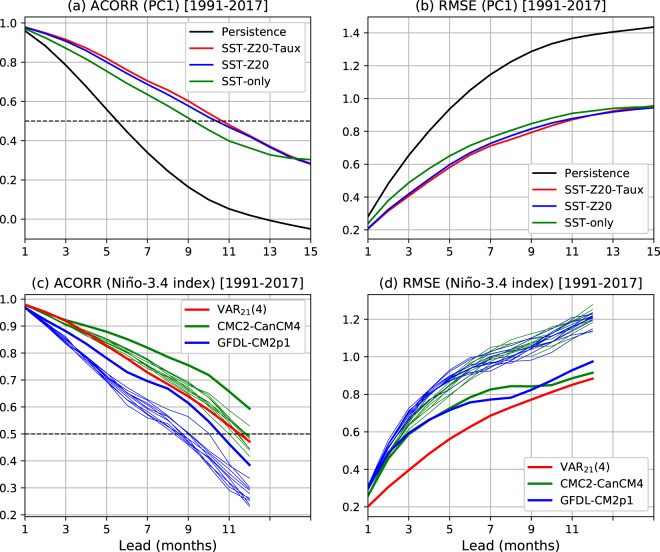
Forecast skills (ACORR and RMSE) as a function of lead time for (**a**,**b**) three VAR models and (**c**,**d**) the best-skilled VAR model (SST-Z20-Taux model) and two dynamical seasonal prediction models from the North American multi-model ensemble (NMME). Skills for SST-PC1 are shown in the top two panels along with the persistence forecast skill (black curves) for comparison. The bottom panels show skills for the Niño-3.4 index from CMC2-CanCM4 (green curves) and GFDL-CM2p1 (blue curves), as well as from the SST-Z20-Taux model (red curves). The thick and thin curves indicate the 10-ensemble mean and member forecast skills, respectively, from the corresponding models. Note that the dynamical model forecasts are available for lead times up to 12 months. The plot was created by python (version 2.7.6) and matplotlib (version 2.2.2) package^37^ (https://www.anaconda.com/distribution/).

### ENSO prediction skills

The forecast skills from the three best-skilled VAR models and the two dynamical seasonal prediction models from NMME are shown in Fig. 3. As expected, all three VAR models perform better than persistence (black curves), showing higher ACORR values and lower RMSE values at all lead times. Between the VAR models, the SST-Z20-Taux model performs the best, followed closely by SST-Z20 model and both these models clearly outperform the SST-only model at all lead times (as expected from Fig. 1). The slightly better skill of the SST-Z20-Taux model, over that of the SST-Z20 model, is evident for lead times up to 11 months for both ACORR and RMSE. Similar comparative performances of the models are also found in the fully cross-validated versions of the models (Supplementary Fig. S2), giving confidence to the results presented above. Note that the relatively small skill differences between the three VAR models should not be interpreted as the insignificance of Z20 and Taux in ENSO dynamics. Rather, the skill improvements are small because the SST-only model, for example, already incorporates much of the Z20 and Taux contributions to observed SST evolutions^27^, as the three variables are (moderately) correlated. A VAR model that includes all three variables is still useful because we can then explicitly model their roles in, and quantify their impacts on, ENSO prediction, in addition to the small skill increase.

An estimate of forecast uncertainty for the VAR models may be obtained by assuming that the model variables (and their linear forecasts) have a multivariate normal distribution^22^. Then the forecast errors will also be normally distributed (as verified in Fig. 2). The 95% confidence limits for SST-PC1 forecasts, calculated under this assumption, are shown for the prominent El Niño and La Niña events occurred during the verification period (Supplementary Fig. S3). It is seen that the forecasted SST-PC1 (shown for the 4-month lead) match well with its observed values, especially for the strong events. We also note that, for all events shown, the observed values are within the 95% confidence intervals of the forecasted values.

Figure 3c,d compare the forecast skills for the Niño-3.4 index from VAR_21_(4), the best of the three VAR models, with those calculated from CMC2-CanCM4 and GFDL-CM2p1 models’ hindcasts/forecasts for the same verification period 1991–2017. These two models represent the top and bottom ends of the NMME models, which exhibit a range of ENSO forecast skills as represented by the Niño-3.4 index^2^. Moreover, the CMC2-CanCM4 ensemble-mean forecast skill matches very well the NMME mean forecast skill; see Barnston *et al*.^2^ for a detailed discussion of the ENSO forecast performance by these and other NMME dynamical models, including the model documentations. Figure 3c,d show the skills from the two dynamical models, along with VAR_21_(4) forecast skills. At short lead times, the VAR_21_(4) model’s forecast skill is similar to the ensemble-mean forecast skill of the better-performing CMC2-CanCM4 model. Beyond three months, the VAR model’s skill falls quicker than that of CMC2-CanCM4 ensemble-mean forecast, but remains comparable to the skills of its individual ensemble members. On the other hand, the VAR_21_(4) forecast skill is superior than that of GFDL-CM2p1, including its ensemble-mean skill.

These analyses confirm the results from many previous studies that empirical models give useful ENSO prediction skills^3,7,8,18,20,21,27^ and, for our best performing VAR model, the Niño-3.4 forecast skill is comparable to that of the best performing NMME dynamical model. The lead time for skilful prediction (or predictability limit) is found to be around 11 months, taking an ACORR value of 0.5 as a measure of skilful prediction^17,28^. This ACORR value occurs at the lead time for which the persistence forecast RMSE becomes comparable to the climatological standard deviation of the observed ENSO index; that is, when the normalised RMSE ~1 (cf. the top panels of Fig. 3). The VAR forecast skill is also comparable to the skill obtained from other empirical models that use different model estimation methods^7,8,18,21^, as well as include ENSO seasonality and/or nonlinearity in their models^7,20,21^. This indicates the robustness of the ENSO prediction limit obtained from the VAR model. One of the limiting factors in extending ENSO prediction skill beyond this limit, at least at present, is the so-called spring predictability barrier, which has been a feature of both empirical and dynamical seasonal forecasts^2,3,21^. Due to the spring predictability barrier, the ENSO prediction skill shows a pronounced seasonal variation. This is also the case in our VAR model forecasts, with the skill increasing to almost 13 months for the target months in boreal spring and decreasing to around 6 months after that (Supplementary Fig. S4). Note that other measures of skilful prediction limit have also been used in the literature. For example, a “predictability horizon” has been used to indicate the time limit at which the skills of CMIP5 (dynamical) decadal forecasts remain “useful”^29^. This is done by comparing the RMSEs of the dynamical forecasts with those of randomly generated “forecasts”.

### Factors affecting ENSO prediction skills

ENSO evolutions are affected by many interacting processes involving forcing, damping and feedbacks of SST, Taux and thermocline depth anomalies in the tropical Pacific^30^, as well as external influences from extratropics and other ocean basins. These ENSO processes can be identified with the elements of submatrices in matrix *A*_0_ and its higher-order counterparts (*A*_1_*, A*_2_*, A*_3_) (refer to Eqs. (1) and (2) in Methods for more details). To investigate their impacts on ENSO prediction skill, we conducted four hindcast experiments by successively eliminating four major processes from the VAR_21_(4) model: (i) total Taux forcing of SSTA, (ii) Taux feedback due to SSTA, (iii) external Taux forcing of SSTA and (iv) the Z20 feedback. Note that this “Z20 feedback” involves only the 2^nd^ EOF mode related to the recharge-discharge of the tropical Pacific WWV. A similar technique was also used by Newman *et al*.^4^ to examine the effect of thermocline variations only, although details of their model and the analysis technique differ from what we have used here.

**Figure 4:**
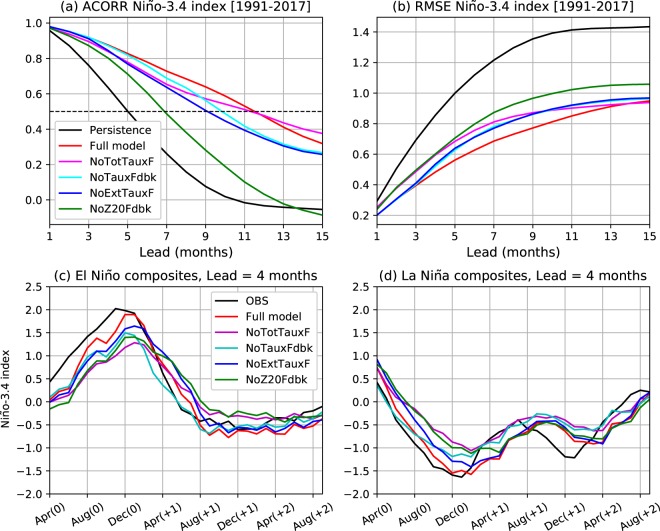
Forecast skills (top panels) and composites of forecast El Niño and La Niña events (bottom panels) computed using the Niño-3.4 index from four experiments, in which the SST-Z20-Taux model is modified by successively eliminating the processes: (i) total zonal wind forcing of SSTA (purple curves), (ii) zonal wind feedback due to SSTA (cyan curves), (iii) external zonal wind forcing of SSTA (blue curves) and (iv) the Z20 feedback (green curves). The submatrices relevant to these processes are: *A*_*TX*_*, A*_*XT*_*, A*_*XX*_, and *A*_*TZ*_, respectively (see Eqs. 1 and 2 in Methods). The skills from the full model (red curves) and persistence (black curves) forecasts are also plotted in the top panels for ease of comparison. The ENSO composites in the bottom panels were made from the four-month lead forecasts of three El Niño events (1997–1998, 2002–2003, 2015–2016) and three La Niña events (1998–1999, 2007–2008, 2010–2011). The observed (black curves) and full model (red curves) composites are also shown for comparisons. The plot was created by python (version 2.7.6) and matplotlib (version 2.2.2) package^37^ (https://www.anaconda.com/distribution/).

The ENSO prediction skills from these experiments are compared with the skills from the full VAR model and persistence forecasts (Fig. 4). It is clear that eliminating the Z20 feedback has the most dramatic impact on ENSO prediction skills, as shown by the smallest ACORR and largest RMSE (except for persistence) at all lead times (green curves). That is, in this case, the *changes* in ACORR and RMSE with respect to the full model are the largest. Given the importance of subsurface processes on ENSO dynamics, it is perhaps not surprising to see the dominant role of Z20 feedback on ENSO prediction skill. This is consistent with the results of previous studies which reported the important contribution from thermocline variations on ENSO prediction skill^4,7,8,21^. Note that the prediction skill without the Z20 feedback is less than that from the SST-only model (cf. Fig. 3), because in the latter model most of the effect of thermocline variations on SST-PC1 was modelled through the included SST-PCs (to the extent they are correlated with Z20-PC2). The next most important process is the total zonal wind forcing (purple curves): eliminating this process results in the second smallest ACORR at short lead times and second largest RMSE for lead times up to 9 months. Eliminating the external zonal wind forcing (caused by the SST-independent part of zonal wind) also substantially reduces the ACORR values; in fact the reduction in this case is more than that results from removing the total zonal wind forcing at lead times of about 5 months and longer (top-left panel, blue curve). However, the RMSE values for the no external zonal wind forcing case remain smaller than those for the no total zonal wind forcing case for lead times up to 9 months (top-right panel), indicating the relatively stronger role of the latter process. Eliminating the zonal wind feedback alone (the SST-dependent part of zonal wind) has the least effect on ENSO predictability (cyan curves): in this case the changes in ACORR and RMSE from those for the full model are the smallest, although the RMSE values nearly match the values for no external zonal wind forcing. The dominance of the total zonal wind forcing over the external zonal wind forcing and the zonal wind feedback may be understood by noting that the latter two processes contribute to the former via the Taux increments (see, e.g., the middle row of matrices A_*i*_ in Eqs. 1 and 2 in Methods).

Despite its well-known role in ENSO dynamics, it is not clear exactly how PC2_Z20_, which is related to WWV anomalies, affects the memory involved in changing the ENSO forecast skill in these full and truncated VAR models. To clarify this, we plot El Niño and La Niña composites, each made from three major observed and hindcast events (at 4-month lead) during our verification period (Fig. 4, bottom panels). The evolutions of the individual El Niño and La Niña events comprising the respective composites may be found in Supplementary Fig. S5. The full model composite (red curve) compares well with the observed composite (black curve) during the decay phase of El Niño (bottom-left panel) and the developing phase of La Niña (bottom-right panel). However, during the El Niño development and La Niña decay phases, there is a time lag between observations and the full model hindcasts. That is, the transitions of ENSO events from their negative to neutral state or from neutral to positive state are delayed. Delayed positive transitions are also seen in many dynamical models, especially at long lead times^2^. The reason for these delayed transitions appears in part to be the spring predictability barrier mentioned before (Supplementary Fig. S4). For example, only the hindcasts starting from March or later tend to develop into El Niño events (recall that the hindcasts are for the 4-month lead). Eliminating the thermocline interaction (green curve) now introduces time lags also for the decay phase of El Niño events and the development phase of La Niña events, thus making the hindcast El Niño and La Niña events somewhat out of phase with those from observations. Eliminating the total zonal wind forcing, on the other hand, reduces the amplitude of both the hindcast El Niño and La Niña events, but doesn’t create any significant phase lag with observations. That is, the thermocline interaction and total zonal wind forcing mostly affect, respectively, the phase and amplitude of the hindcast ENSO events. The phase shift both decreases correlation coefficient and increases RMSE, whereas the reduced amplitude mostly increases RMSE only. This explains the largest reduction in ENSO hindcast skill due to the elimination of thermocline interaction.

## Summary and Discussion

We have developed and used a fourth-order multi-variable VAR model of tropical Pacific atmosphere-ocean interactions to investigate the factors affecting the model’s ENSO forecast skill. The model makes skilful ENSO forecasts for up to 11 months lead, as measured by the anomaly correlations and RMSEs of both SST-PC1 and the Niño-3.4 index. This skill is shown to be comparable to the ensemble-mean forecast skill of the best performing NMME model (CMC2-CanCM4) at short lead times (although at longer lead times the empirical model falls behind this dynamical model). The VAR model skill is also comparable to the published skills from other empirical models that use different model estimations methods and include features like ENSO seasonality and nonlinearity. The fourth-order VAR model used in this study has higher ENSO prediction skill than its first-order and SST-only counterparts have. This indicates that for most skilful ENSO forecasts, at least by empirical models, the immediate past evolutions of the variables are also important in addition to their latest state, as reported in other empirical model studies.

The VAR model’s high ENSO forecast skill and its relative simplicity allowed us to examine the effects of major forcing and feedback processes on its ENSO forecast skill. We find that the most important of the processes is the thermocline feedback, which increases the ENSO forecast skill by over four months in our linear model. The other ENSO processes, with successively smaller impacts on the forecast skill, are the total zonal wind forcing of SST, the external zonal wind forcing and the zonal wind feedback due to SST, although the relative importance of the last two processes is less certain (having similar RMSEs; Fig. 4). The mechanisms by which the two most influential processes affect ENSO predictability in the VAR model are, however, different. The thermocline feedback improves the forecast skill by maintaining the correct phase, whereas the total zonal wind forcing improves the skill by maintaining the correct amplitude of the forecast ENSO events.

These results, although obtained from an observation-based empirical model, may be useful for understanding the causes of variations in ENSO forecast skill in dynamical seasonal prediction models^2^. As in nature, the memory associated with ENSO evolutions is determined by the mean state and various forcings and feedbacks simulated by the models. Biases in these processes are likely to affect the models’ ENSO forecast skills, and the processes that dominate a model’s ENSO dynamics are more likely to affect the model’s forecast skill. Indeed, the ENSO forecast skill of an APEC Climate Center seasonal forecast model was found to be adversely affected by the mean state bias caused by thermocline feedback errors at long lead times^31^. Here, we have used observations and reanalyses to derive the empirical model used in the diagnosis of the contributions from different processes. However, this approach can as well be applied to long control simulations of dynamical models, especially those used for seasonal predictions. In this case, one can train an empirical model on a section of the control simulation, examine the model’s goodness-of-fit and then perform “forecast” experiments by eliminating different important processes one at a time, as we did in this study. Of course, the linear empirical model will not fully capture the nonlinear dynamics of ENSO; however, much can still be learned about this important phenomenon by studying its linear dynamics.

## Methods

### Data and preliminary processing

The SST, Taux and Z20 data used for model fitting and verification were obtained from the HadISST^32^, ERA-40/ERA-Interim^33,34^ and the Australian Bureau of Meteorology’s POAMA Ensemble Ocean Data Assimilation System (PEODAS)^35^, respectively. A common time period (1960–2017) over which all three variables are available was chosen for our analysis. The Taux was taken from both ERA-40 (for the period 1960–1978) and ERA-Interim (1979–2017). First, the non-seasonal anomalies were calculated by removing from the monthly-mean variables the respective time means and climatological annual cycles at each grid point. The anomalies were then divided by their respective domain standard deviations before being subjected to separate empirical orthogonal function (EOF) analysis over the tropical Pacific domain (130E-80W, 20S-20N). The EOFs (Supplementary Fig. S1) were computed over 1960–1990, which is the training period for our empirical models (see below). The corresponding principal component (PC) time series were obtained by projecting the SST, Taux and Z20 anomalies for the entire period (1960–2017) onto the respective EOF sets. The linearly detrended PCs were then used to build the VAR models and verify model forecasts, as discussed below.

### Models

An *m*-component VAR model of order *p*, VAR_*m*_(*p*), may be written as^22^:

1 $${{\bf{X}}}_{t+1}={\sum }_{i=0}^{p-1}{A}_{i}{{\bf{X}}}_{t-i}+{{\bf{W}}}_{t+1};\,p=1,2,3,\ldots $$

Here **X** is an *m*-component state vector: [PC1_SST_, PC2_SST_, …, PC1_Taux_, PC2_Taux_,…, PC1_Z20,_ PC2_Z20_, …]^T^, *t* is the month index, A_*i*_ are *m* × *m* system matrices (containing forcing, damping and feedbacks) and **W** is a residual noise vector (temporally white, but spatially correlated). The state vector consists of the linearly detrended PC time series of SST, Taux and Z20. The PCs are centered, so an intercept term doesn’t appear in Eq. (1). The system matrices were estimated by the least squares method. More details of a *p*-order multi-variable VAR model and its parameter estimation may be found in existing literature^22,23^.

The first-order (*p* = 1) model (**X**_*t+1*_ = A_0_ **X**_*t*_ + **W**_*t+1*_) may be written as:

2 $$\mathop{\underbrace{[\begin{array}{c}{\boldsymbol{P}}{{\boldsymbol{C}}}_{SST}\\ {\boldsymbol{P}}{{\boldsymbol{C}}}_{Taux}\\ {\boldsymbol{P}}{{\boldsymbol{C}}}_{Z20}\end{array}]}}\limits_{{\bf{X}}}(t+1)=\mathop{\underbrace{[\begin{array}{ccc}{A}_{TT} & {A}_{TX} & {A}_{TZ}\\ {A}_{XT} & {A}_{XX} & {A}_{XZ}\\ {A}_{ZT} & {A}_{ZX} & {A}_{ZZ}\end{array}]}}\limits_{{{\rm{A}}}_{0}}\mathop{\underbrace{[\begin{array}{c}{\boldsymbol{P}}{{\boldsymbol{C}}}_{SST}\\ {\boldsymbol{P}}{{\boldsymbol{C}}}_{Taux}\\ {\boldsymbol{P}}{{\boldsymbol{C}}}_{Z20}\end{array}]}}\limits_{{\bf{X}}}(t)+\mathop{\underbrace{[\begin{array}{c}{{\boldsymbol{W}}}_{SST}\\ {{\boldsymbol{W}}}_{Taux}\\ {{\boldsymbol{W}}}_{Z20}\end{array}]}}\limits_{{\bf{W}}}(t+1)$$

The elements in matrix A_0_ are submatrices representing self and mutual interactions of the state variables, with the submatrix suffixes *T*, *X*, and *Z* denoting SST, Taux and Z20, respectively. For example, the elements of submatrix *A*_*TT*_ represent the damping of and interactions between the included SST modes, whereas the elements of submatrix *A*_*TX*_ represent the forcing of SST modes by Taux modes. The other submatrices may be interpreted in a similar fashion. The size of these submatrices may be different, depending on the number of PCs retained for each variable (determined in Model selection subsection). For a *p*-order VAR model, there are *p* number of matrices A_*i*_ (corresponding to state vectors **X**_*t−i*_), each having the same structure as A_0_.

### Model retrospective forecasts and verification

Tropical Pacific SST, Taux and Z20 anomalies for 1960–2017 were divided into two subsets: a 31-year (1960–1990) training dataset and a 27-year (1991–2017) verification dataset. All the models discussed in the paper were fitted using the training dataset and verified against the verification dataset. Retrospective forecasts (or hindcasts) were made for lead times up to 15 months with one-month time steps, starting from each month during 1991–2017. ENSO forecast skills were measured by anomaly correlation (ACORR) and root-mean-squared-error (RMSE) of PC1_SST_ and the Niño-3.4 index; the latter for comparisons with other published results. The Niño-3.4 index was calculated by first transforming the forecast SST-PCs back to the physical space and then averaging the resulting SSTs over the Niño-3.4 region (170W-120W, 5S-5N). This EOF-filtered forecast Niño-3.4 index was, however, verified against the full observed Niño-3.4 index. Additionally, a fully cross validated forecast verification was also done over the entire data period (1960–2017) by repeatedly withholding data from a 15-month sliding window, fitting the model on remaining data and computing forecast skills using the withheld data. The EOFs were re-calculated during each repetition from the relevant model training data to ensure full cross-validation.

To compare the VAR model’s ENSO prediction skill with those from dynamical models, we also analysed seasonal-to-interannual predictions by two representative dynamical models from the North American multi-model ensemble (NMME)^36^. The 10-member ensemble hindcast/forecast data from these models were processed to calculate the Niño-3.4 index prediction skills. The SST anomalies were first calculated by subtracting the model climatology (a function of forecast lead times, as well as the calendar months) at different grid points from the respective model’s forecasts. Subtracting the model climatology from forecast fields separately at each lead time should remove any model drifts. However, the GFDL model forecast anomalies still contained some drift, which was removed by linear detrending. Then the ACORR and RMSE skills were calculated from these anomalies using the same method and verification period as for the VAR model.

## Supplementary information

Supplementary Information.
