# Supplementary material for: Factors affecting ENSO predictability in a linear empirical model of tropical air-sea interactions
Source: Sci Rep. 2020 Mar 3;10:3931. doi: 10.1038/s41598-020-60371-1 (PMC7054313; doi:10.1038/s41598-020-60371-1)
Supplement: Supplementary file 1 — Supplementary Information. [file 41598_2020_60371_MOESM1_ESM.pdf]

Factors affecting ENSO predictability in a linear empirical model of tropical air-sea interactions

Harun A. Rashid

Climate Science Centre, CSIRO Oceans and Atmosphere, Aspendale, VIC 3195, Australia
Email address: Harun.Rashid@csiro.au

Supplementary Information

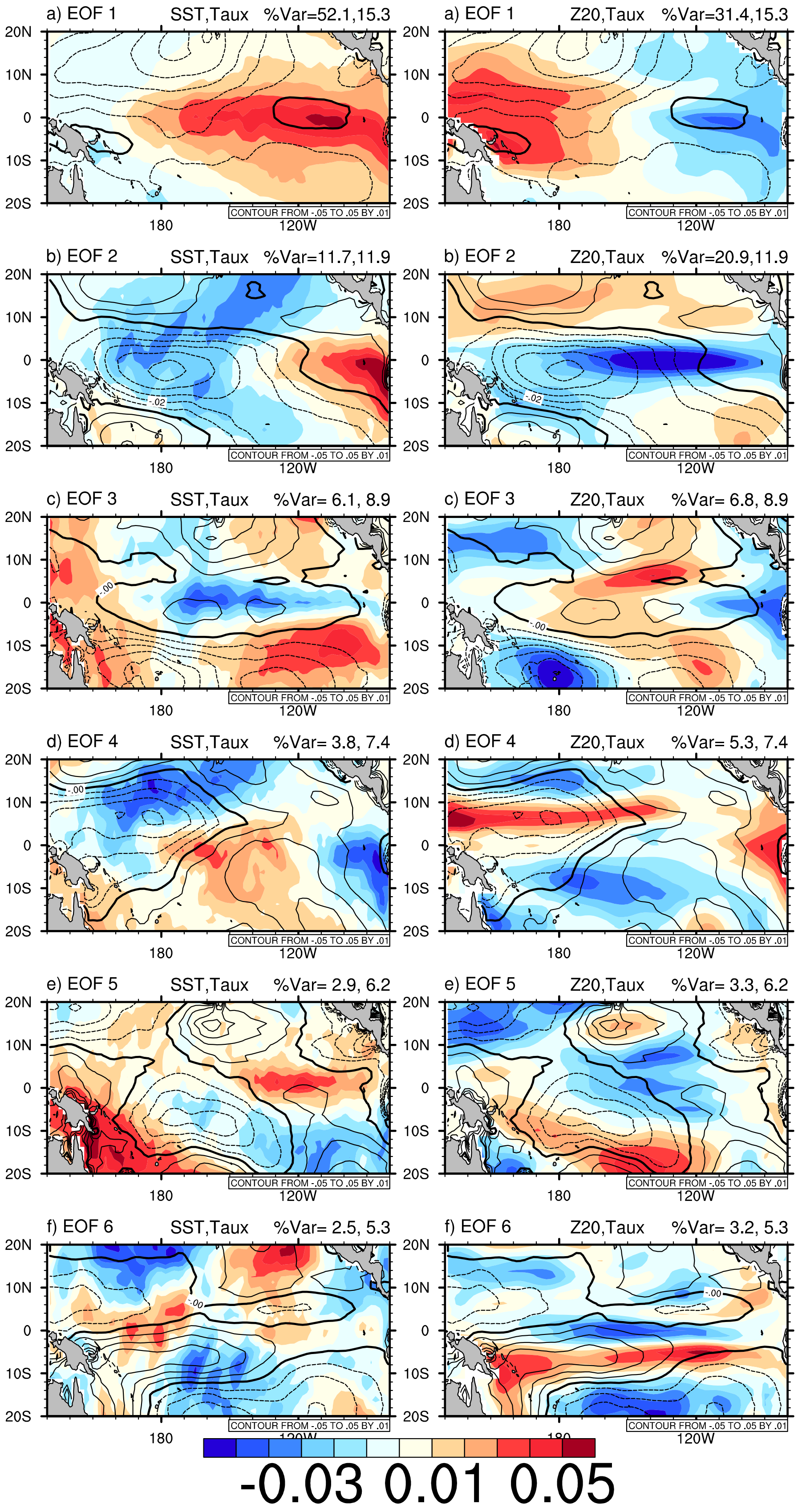

Figure S1. Normalized spatial patterns of the six leading EOFs of observed SST (left column) and Z20 (right column) anomalies over a common period of 1960-1990; the EOFs of the ZWS anomalies are overlaid as contours on both the SST and Z20 EOFs. The plot was created by NCAR Command Language, version 6.3.0 (http://ncl.ucar.edu/).

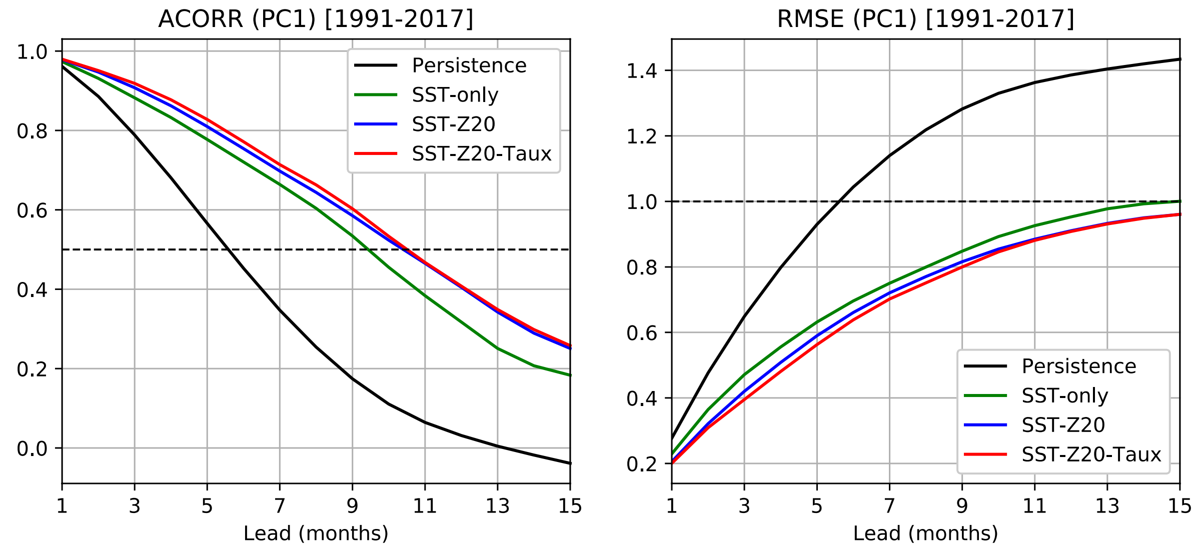

Figure S2. As in Figures 3a,b, except for the forecast skills are now obtained from the fully cross validated forecast verifications. In this approach, the VAR model was trained over the remaining data for 1960-2017 after repeatedly withholding 15-month of data from a sliding window and verified on the withheld data. The forecast skills for 1991-2017 are shown to compare with those in Figures 3a,b. The plot was created by python (version 2.7.6) and matplotlib (version 2.2.2) package[34] (https://www.anaconda.com/distribution/).

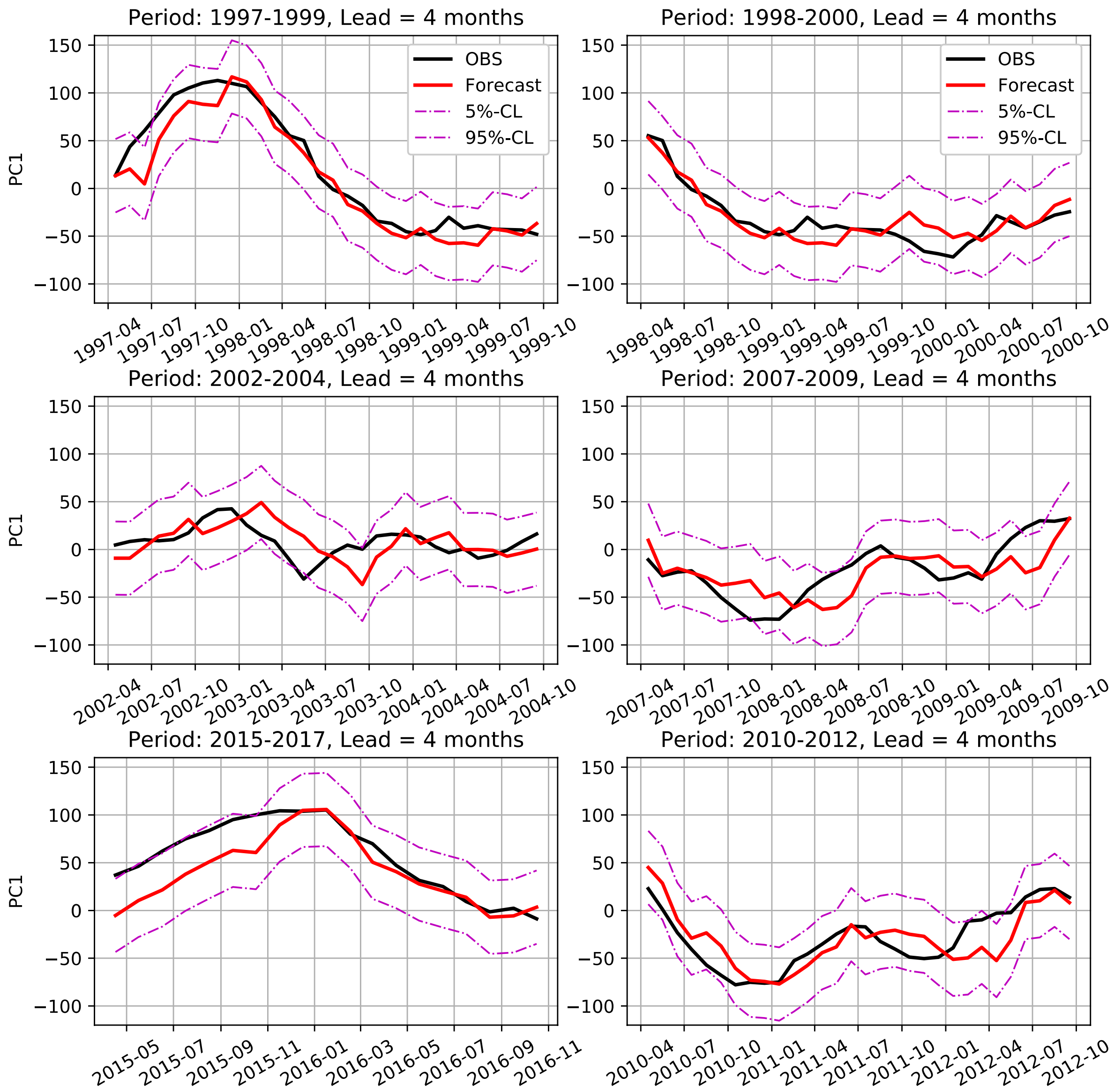

Figure S3: Forecast SST-PC1 (4-month lead) for the prominent El Niño and La Niña events (red curve) during the verification period 1991-2017 and their 5-95% confidence limits (magenta curves). The observed PC1 evolution is also shown for each event (black curve). The observations and forecasts, from the SST-Z20-Taux model, are shown for three El Niño events (1997-1998, 2002-2003, 2015-2016) and three La Niña events (1998-1999, 2007-2008, 2010-2011). The plot was created by python (version 2.7.6) and matplotlib (version 2.2.2) package[34] (https://www.anaconda.com/distribution/).

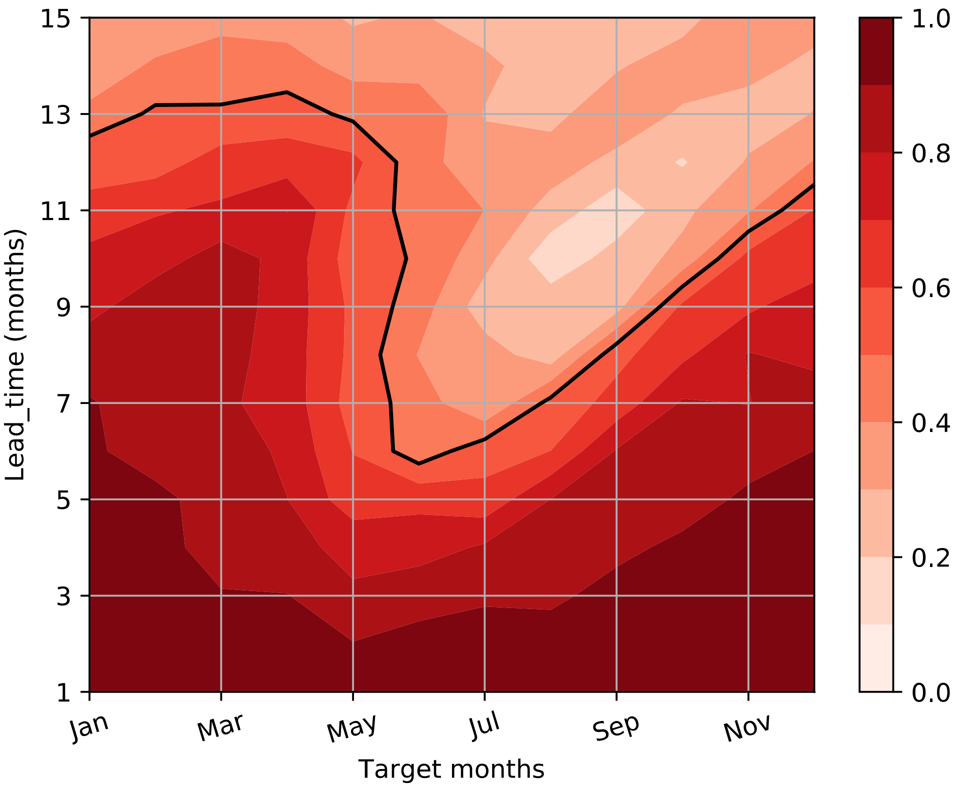

Figure S4. Seasonal variation of ENSO forecast skill (ACORR), as a function of lead time and target month, from the SST-Z20-Taux model. The training and verification periods are 1960-1990 and 1991-2017, respectively (as for Fig. 3). The black solid line indicates ACORR = 0.5. The forecast skills for target months after the boreal spring decline dramatically, as a result of the well-known spring predictability barrier. The plot was created by python (version 2.7.6) and matplotlib (version 2.2.2) package[34] (https://www.anaconda.com/distribution/)

.

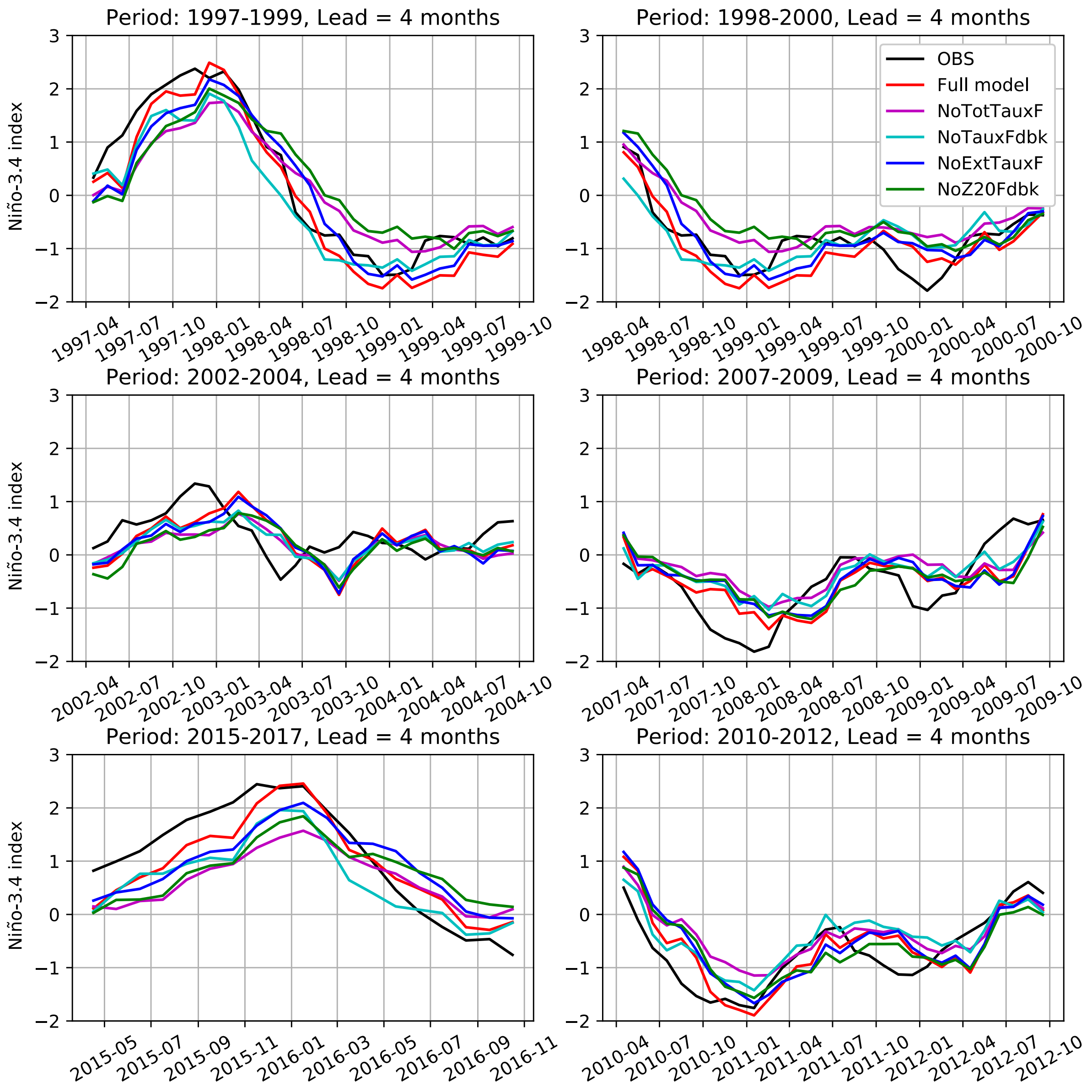

Figure S5. Temporal evolutions of El Niño and La Niña events in 4-month lead forecasts of SST-PC1 from four experiments, in which the SST-Z20-Taux model is modified by successively eliminating the processes: i) total ZWS forcing of SSTA (purple curves), ii) ZWS feedback due to SSTA (cyan curves), iii) external ZWS forcing of SSTA (blue curves) and iv) the Z20 feedback (green curves). Shown are the evolutions of three El Niño events (1997-1998, 2002-2003, 2015-2016) and three La Niña events (1998-1999, 2007-2008, 2010-2011). The corresponding events from observations (black curves) and the full model forecasts (red curves) are also shown for comparisons. The plot was created by python (version 2.7.6) and matplotlib (version 2.2.2) package[34] (https://www.anaconda.com/distribution/).
